# Deep Learning Predicts Correlation between a Functional Signature of Higher Visual Areas and Sparse Firing of Neurons

**DOI:** 10.3389/fncom.2017.00100

**Published:** 2017-10-30

**Authors:** Chengxu Zhuang, Yulong Wang, Daniel Yamins, Xiaolin Hu

**Affiliations:** ^1^Tsinghua National Laboratory for Information Science and Technology, Department of Electronic Engineering, Tsinghua University, Beijing, China; ^2^Department of Psychology, Stanford University, Stanford, CA, United States; ^3^Tsinghua National Laboratory for Information Science and Technology, Department of Computer Science and Technology, Tsinghua University, Beijing, China; ^4^Computer Science Department, Stanford Neurosciences Institute, Stanford University, Stanford, CA, United States; ^5^Center for Brain-Inspired Computing Research, Tsinghua University, Beijing, China

**Keywords:** visual processing, deep learning, higher-order statistics, V1, V2, V4

## Abstract

Visual information in the visual cortex is processed in a hierarchical manner. Recent studies show that higher visual areas, such as V2, V3, and V4, respond more vigorously to images with naturalistic higher-order statistics than to images lacking them. This property is a functional signature of higher areas, as it is much weaker or even absent in the primary visual cortex (V1). However, the mechanism underlying this signature remains elusive. We studied this problem using computational models. In several typical hierarchical visual models including the AlexNet, VggNet, and SHMAX, this signature was found to be prominent in higher layers but much weaker in lower layers. By changing both the model structure and experimental settings, we found that the signature strongly correlated with sparse firing of units in higher layers but not with any other factors, including model structure, training algorithm (supervised or unsupervised), receptive field size, and property of training stimuli. The results suggest an important role of sparse neuronal activity underlying this special feature of higher visual areas.

## Introduction

After a complex visual pattern enters the visual system of mammals, the pattern undergoes different processing stages. In general, each stage captures the pattern in different abstraction levels. For instance, many neurons in the primary visual cortex (V1) are sensitive to edges (Hubel and Wiesel, 1962, 1968), some neurons in the visual area V2 are sensitive to line conjunctions or corners (Hegde and Van Essen, 2000; Ito and Komatsu, 2004), and some neurons in the inferior temporal cortex are sensitive to the whole pattern, such as faces or cars (Kanwisher et al., 1997; Epstein and Kanwisher, 1998; Gauthier et al., 2000). But the differences among the simple response properties of neurons in various areas are not always prominent and robust. For example, the neural responses to many artificial stimuli in V2 are largely similar to those in V1 (Peterhans and Vonderheydt, 1989; Hegde and Van Essen, 2000; Lee and Nguyen, 2001).

Using controlled naturalistic texture stimuli, electrophysiological recordings revealed that neurons in macaque V2 (Freeman et al., 2013) and V4 (Okazawa et al., 2015) but not V1 prefer stimuli with the higher-order statistical dependencies found in natural images rather than in spectrally matched noise stimuli that lack naturalistic structures. Consistent with this, functional magnetic resonance imaging measurements in humans demonstrated a much higher preference for stimuli with naturalistic higher-order statistics in V2, V3, and V4 than in V1 (Freeman et al., 2013). These results suggest that the sensitivity to naturalistic textures is a functional signature of higher areas of the visual cortex. However, it remains unknown how this signature emerges.

Because the naturalistic texture images used in these experiments (Freeman et al., 2013; Okazawa et al., 2015) were synthesized by matching various higher-order dependencies among linear and energy filters (akin to V1 simple and complex cells, respectively) to those present in natural images, it is straightforward to assume that higher areas encode correlations among the output of V1 neurons. Given this assumption, a hierarchical model in which higher layers take the combined efferents of lower layers as afferent would exhibit a functional difference similar to that found between V1 and higher areas (Freeman et al., 2013). However, the principles underlying a model built to lead to this difference are unknown. Simply stacking a computational module one by one with random connections between them is likely insufficient (see section Higher Layer Units Prefer Naturalistic Texture Images). It is also unknown what factors in the models will contribute to the difference and how they will contribute. Answers to these questions may shed light on how the functional signature emerges in higher visual areas.

In the present study, we first discovered that the signature is a common property in the higher layers of several hierarchical deep learning models (Krizhevsky et al., 2012; Hu et al., 2014; Simonyan and Zisserman, 2015), which are built on the extended theory of V1 simple and complex cells (Hubel and Wiesel, 1962, 1968) in higher areas. Although quite different in learning principles, either for achieving high classification accuracy or for achieving good reconstruction of the input, after training, the higher layer units in these models were found to be more sensitive to the synthetic naturalistic images containing higher-order statistical dependencies than to spectrally matched noise that lack them. By contrast, only a weak though significant preference was observed in lower layer units. A positive correlation was demonstrated between the strength of this signature and the sparseness of responses in higher layer neurons, which suggests that sparse firing may underlie the emergence of the functional signature of higher areas found in primates (Freeman et al., 2013; Okazawa et al., 2015).

## Results

### Stimuli and models

**Figure 1:**
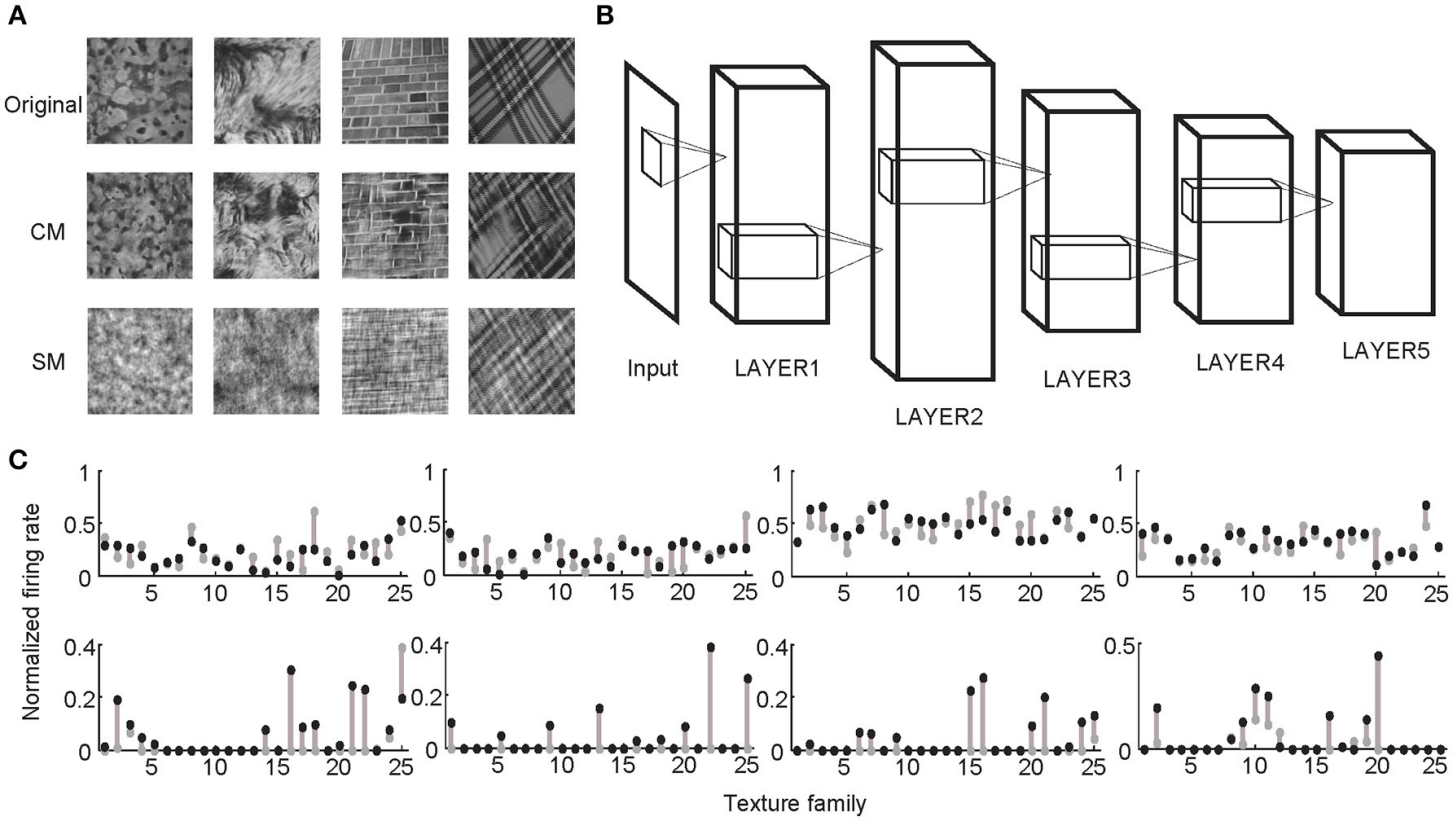
Stimuli and experimental protocol. **(A)** Example stimuli. Based on each natural texture image (top) a pair of CM (middle) and SM (bottom) images was synthesized. **(B)** Illustration of a deep learning model with five big layers, LAYER1 to LAYER5. Each big layer contained at least a convolutional layer (for CNN) or sparse coding layer (for SHMAX), a pooling layer, and sometimes a normalization layer. After LAYER 5, CNN often had several fully connected layers and an output layer for classification, which are not shown here. **(C)** Normalized responses of four sample units in LAYER1 (top) and LAYER5 (bottom) of AlexNet averaged over images in each of 25 texture families. The response of a unit was normalized by dividing its maximum response to all CM images and SM images. The gray dots and black dots denote the responses to SM images and CM images, respectively. Clearly, the LAYER1 units fired more vigorously than the LAYER 5 units.

Following the procedures described in previous studies (Freeman et al., 2013; Okazawa et al., 2015), two sets of synthetic stimuli were generated based on the properties of natural texture images (Figure 1). The first set of stimuli was obtained by randomizing phases of Fourier components in the original images. Therefore, they had the same spectral properties as the original images and were called spectrally matched (SM) images (Figure 1A). The second set of stimuli was generated from Gaussian noise using an iterative procedure, with the aim to match the higher-order statistics in them (correlations between filter responses as well as their energies) to those in the original images. These images were called correlation-matched (CM) images, and they looked similar to the original images as judged by human observers (Portilla and Simoncelli, 2000) (Figure 1A). Based on each natural texture image, respective SM and CM images were synthesized. A total of 25 families of natural texture images (40 per family) were used, yielding 1,000 SM images and 1,000 CM images. Different families of natural texture images had different higher-order statistical dependencies and therefore different appearances, as did different families of CM images (Figure 1A).

Since Hubel and Wiesel discovered simple and complex cells in the V1 area of cats in the 1960s (Hubel and Wiesel, 1962), various computational models for the visual system have been proposed (Fukushima, 1980; LeCun et al., 1989, 1998; Riesenhuber and Poggio, 1999; Ullman, 2007; Hu et al., 2014), and these fall into two categories, supervised and unsupervised learning models. Among those in the first category, the convolutional neural network (CNN) (LeCun et al., 1989, 1998), which showed remarkable performance in a variety of visual recognition and detection tasks (Krizhevsky et al., 2012; LeCun et al., 2015), was selected for investigation in this study. Among those in the second category, we selected the sparse HMAX (SHMAX) model (Hu et al., 2014), which is essentially a hierarchical sparse coding model, an extension of the original biological-inspired model HMAX (Riesenhuber and Poggio, 1999; Serre et al., 2005). Both CNN and SHMAX are capable of learning low-, mid-, and high-level representations of object (Hu et al., 2014; Zeiler and Fergus, 2014), making these models good candidates for this investigation because different levels of representation of visual input have long been known to exist in the ventral stream of the visual cortex (Hubel and Wiesel, 1962; Kanwisher et al., 1997; Epstein and Kanwisher, 1998; Gauthier et al., 2000; Hegde and Van Essen, 2000; Ito and Komatsu, 2004).

Two typical CNNs, AlexNet (Krizhevsky et al., 2012) and VggNet (Simonyan and Zisserman, 2015), were trained on a very large dataset containing millions of images (Russakovsky et al., 2015). SHMAX was trained on a subset of this dataset. Because the models had different numbers of layers, for convenience, some layers were grouped based on their structural properties so that all of the models contained five big layers, LAYER1 to LAYER5 (Figure 1B; section Materials and Methods). These were the main locations in the models we investigated.

### Higher layer units prefer naturalistic texture images

Two sets of stimuli, CM images and SM images, were presented to the three deep learning models, AlexNet, VggNet, and SHMAX, and the responses of each unit in these models were recorded (Figure 1). For each unit, a modulation index between −1 and 1 was calculated to reflect its preference for CM images or SM images (see section Materials and Methods). A modulation index approaching 1 indicates higher preference for CM images, approaching −1 indicates higher preference for SM images, and near zero indicates little preference for either type of stimulus. The mean modulation index of a set of units was defined as the population modulation index (PMI).

**Figure 2:**
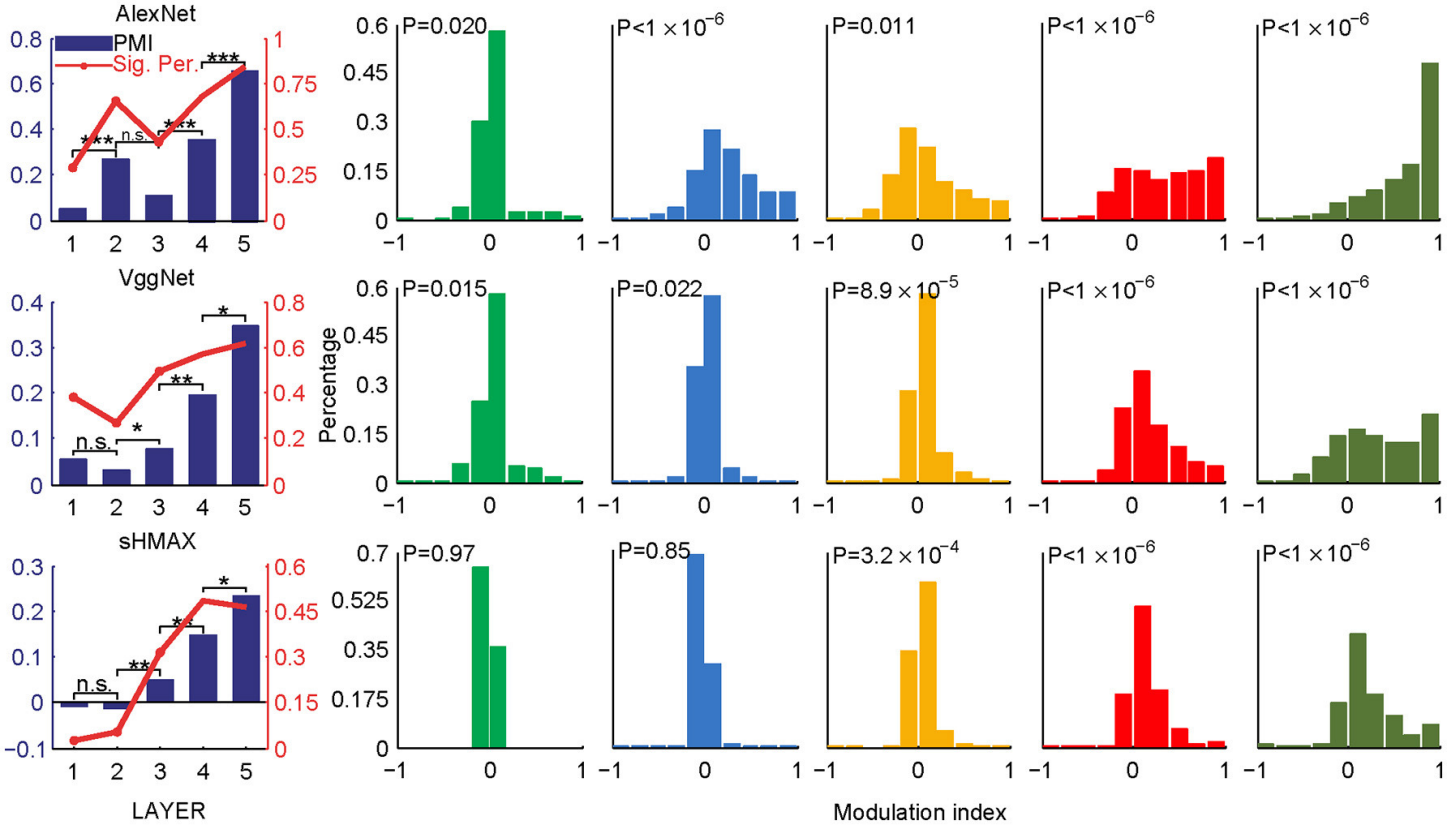
PMIs in different layers of AlexNet (top), VggNet (middle), and SHMAX (bottom) calculated based on all CM and SM images. The bar plots in the first column show the PMI in each layer. The asterisks indicate the results of pairwise random sampling *t*-tests (RST; see section Materials and Methods) determining whether the modulation in any layer is significantly larger than that in the preceding layer (^*^P < 0.05; ^**^P < 0.01; ^***^P < 0.001; n.s., not significant). The solid lines in the first column indicate the percentages of units that show significant positive modulation indices (*P* < 0.05, randomization test for each unit, see section Materials and Methods). The other columns show the distributions of the units in each layer with different mean modulation indices. The *P*-values are averaged *P*-values over 500 RSTs examining whether the modulation was significantly larger than zero (see section Materials and Methods).

The PMIs in LAYER1 in all networks, as well as LAYER2 in VggNet and SHMAX, were close to zero (Figure 2), indicating little preference of these low-level units for any type of image. By contrast, the PMIs in higher layers of these networks were substantially larger than zero (Figure 2). Some units in these layers responded to CM images only (modulation index equaled 1). The PMIs in LAYER4 and LAYER5 were significantly larger than that in LAYER1 (*P* < 10^−5^, unpaired one-tailed *t*-test after repeatedly sampling 100 units from two groups; see section Materials and Methods for details). These results are consistent with findings in primates (Freeman et al., 2013; Okazawa et al., 2015). Moreover, from LAYER2 to LAYER5, there was a general trend for the modulation index to be larger in a layer than in the layer just below it (*P* < 0.05, unpaired one-tailed *t*-test after repeatedly sampling 100 units from two groups; Figure 2, leftmost), except in AlexNet, where the PMI in LAYER3 was smaller than that in LAYER2.

**Figure 3:**
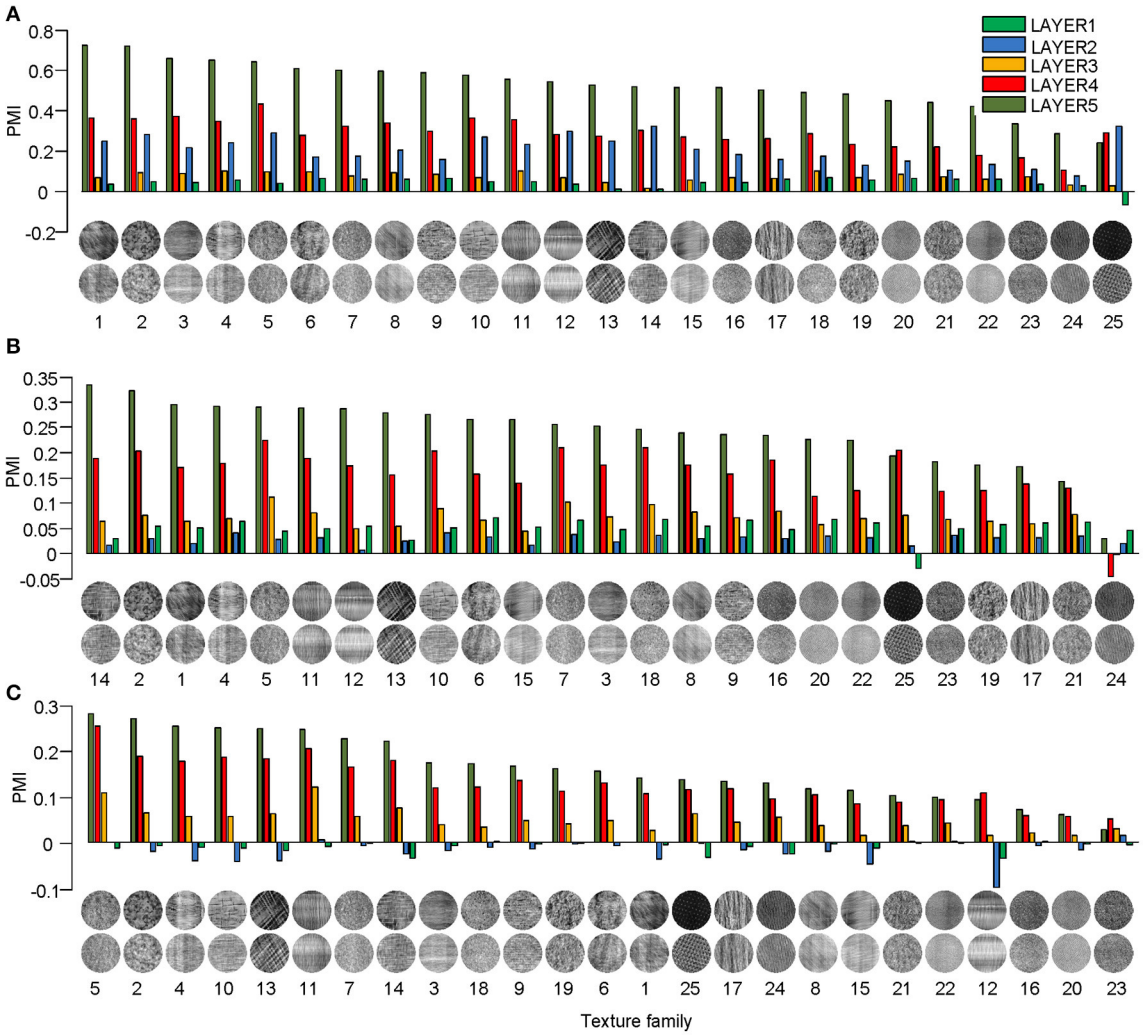
PMIs in each layer of AlexNet **(A)**, VggNet **(B)**, and SHMAX **(C)** calculated separately based on 25 texture families. The texture families shown below are sorted in decreasing order of PMI in LAYER5. The number below each texture family is the rank of that family in the sorted family sequence based on AlexNet.

Different texture families evoked different degrees of response preference to naturalistic structures. We sorted the texture families based on the PMI in the top layers of the three models (Figure 3) and found that the orders were consistent across the models as measured by ranking distance (RD) (section Materials and Methods). The RD-values between the orders of AlexNet and VggNet, between AlexNet and SHMAX, and between VggNet and SHMAX were 11.01, 13.99, and 13.24, respectively. According to a permutation test, these values indicate significant consistency between the orders (*P* = 0.0002, 0.0037, 0.0019, respectively).

**Figure 4:**
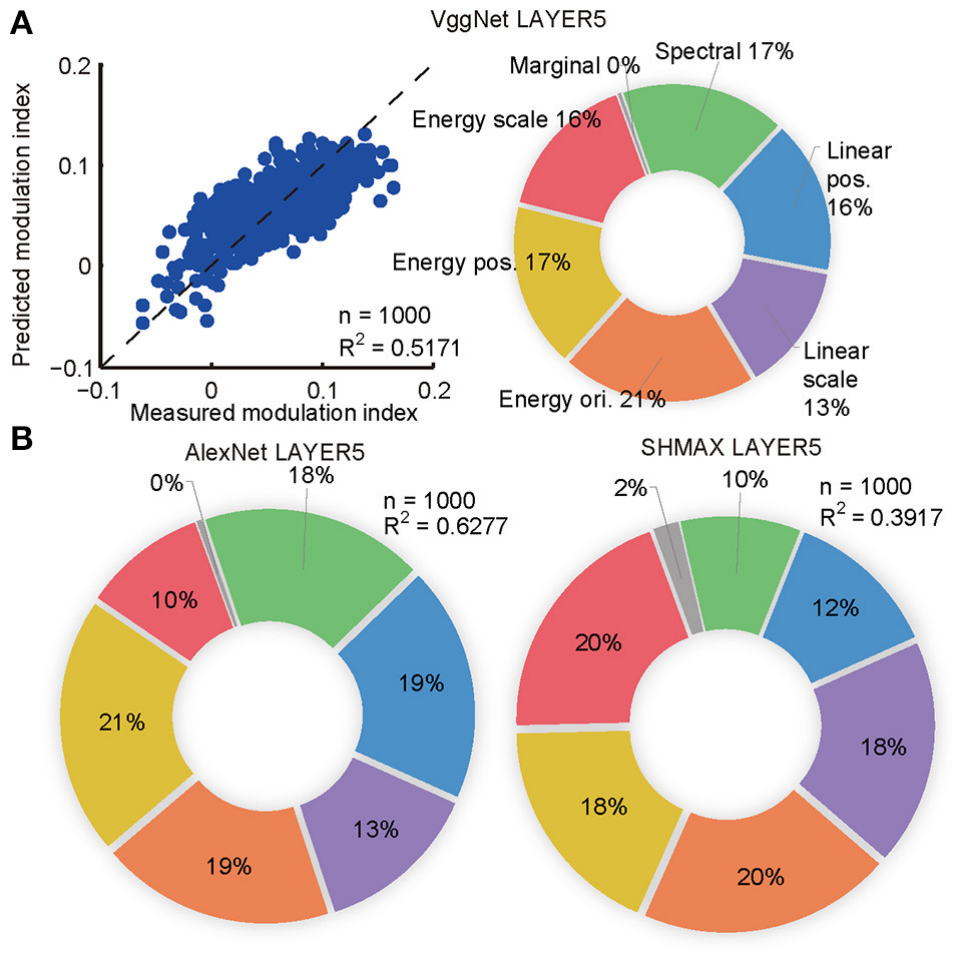
Contributions of different groups of statistics to modulation in the top layers of the models calculated using the averaging-over-orderings technique based on linear regression analysis. **(A)** The left panel shows the result of regression for PMI in LAYER5 of VggNet based on different groups of statistics (correlations of linear or energy filter responses cross-scale, cross-position, and cross-orientation together with marginal and spectral statistics) in the original natural texture images. Each dot represents the measured and predicted PMI corresponding to one pair of CM and SM images. The right panel shows the contributions of different groups of statistics to PMI in LAYER5 of VggNet. **(B)** The contributions of different groups of statistics to PMIs in LAYER5 of AlexNet (left) and SHMAX (right).

The synthesized CM images contained many groups of statistics, including cross-scale, cross-position, and cross-orientation correlations of linear filter responses or energies (L2-norm of responses of two identical linear filters at the same position, scale, and orientation, but differing by 90° in phase). We found that the relative contributions of these statistics to the modulation indices of the top layer units in the models were qualitatively similar to their contributions to human sensitivity (Freeman et al., 2013) and the macaque V4 neuron sensitivity (Okazawa et al., 2015) to synthetic texture images (section Materials and Methods; Figure 4).

**Figure 5:**
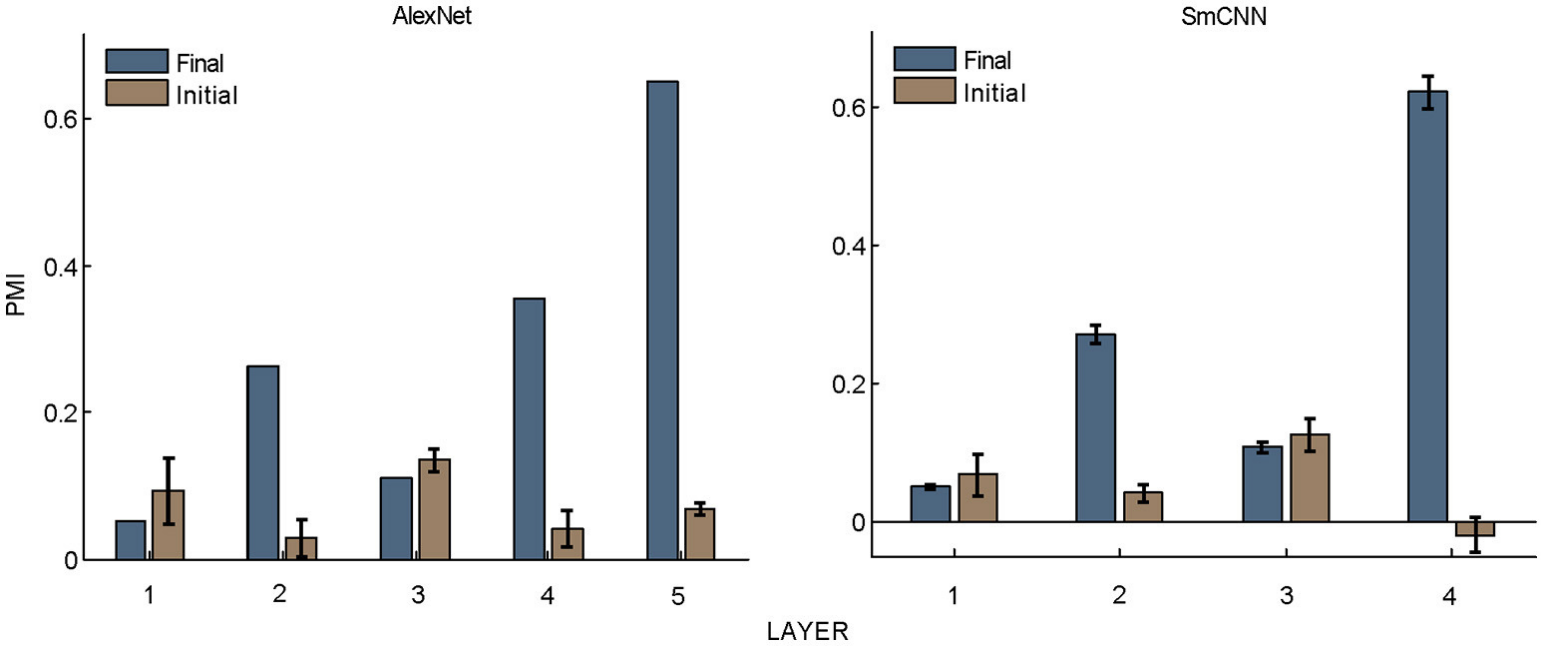
PMIs in each layer of AlexNet and SmCNN before and after training. Error bars indicate the standard deviation of 10 models with the same architecture using different initial values. The results of AlexNet after training are the same as those in Figure 2.

What causes the preference of higher layer units to naturalistic structures in these models? The answer to this question may shed light on the understanding of the mechanism underlying the functional and perceptual signatures of the higher areas in the visual cortex. First, hierarchical structure should play an important role, as it is a property common to all models as well as to the visual cortex. However, this is not the only factor, because models with random weights did not exhibit this signature (Figure 5). Learning should also contribute, and this contribution may not be restricted to specific learning rules because both supervised and unsupervised learning led to similar results. The resolution may lie in the common features of the learning procedures, and these were thus investigated in detail, as described below.

### Response sparseness correlates with modulation

**Figure 6:**
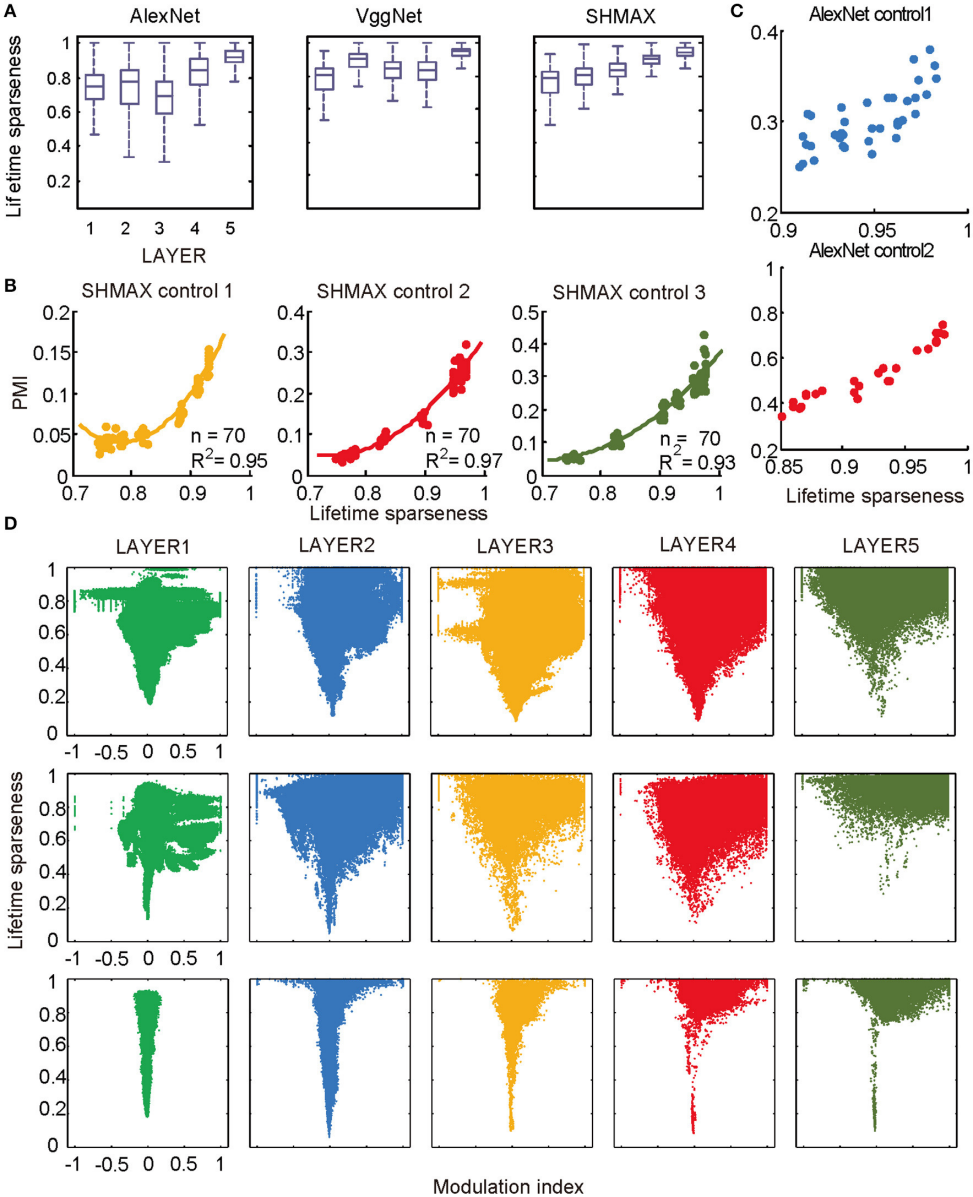
Relationship between the modulation and the response sparseness of units in the models. **(A)** Boxplots of lifetime sparseness in different layers of AlexNet, VggNet, and SHMAX. **(B)** PMI vs. mean lifetime sparseness of all units in three higher layers of SHMAX in three control experiments. Each dot represents the result with a particular λ value in the corresponding layer. The solid curves are quadratic fitting. **(C)** PMI vs. lifetime sparseness of all units in two layers of AlexNet in two control experiments, with correlation *r* being 0.75 and 0.95, respectively. **(D)** Scatter plots of the modulation index and lifetime sparseness of all units in different layers of VggNet (top), AlexNet (middle), and SHMAX (bottom). Each dot represents one unit in the corresponding layer.

We observed different response patterns of units in different layers of the models (Figure 1C), which motivated us to inspect the unit response pattern first. We found that all units in the models exhibited a certain level of response sparseness as quantified using lifetime sparseness (see section Materials and Methods) (Willmore et al., 2011) (Figure 6A). This result was not unexpected for SHMAX because its learning principle encourages the sparse activity of hidden units [see Equation (1) in section Materials and Methods]. The more interesting finding was that the two CNNs also exhibited sparse firing, even though this property was not explicitly specified in their learning rules. Similar results were obtained in a recent study (Yu et al., 2016). It is partly due to the rectified linear function used in these models. Comparison of Figure 6A and Figure 2 suggests a certain amount of correlation between sparseness and modulation. For instance, the top layer of each model had both the highest sparseness and modulation, and both modulation and sparseness increased with ascending layers in SHMAX. Note that the correlations in the two CNNs were not introduced by layer grouping because similar results could be obtained based on the original layers in the models (Figure S1).

We then controlled sparseness for SHMAX and AlexNet to further inspect the relationship between sparseness and modulation. By varying the λ parameter during training of SHMAX [Equation (1) in section Materials and Methods] and AlexNet [Equation (4) in section Materials and Methods], we could separately control the sparseness level of each layer. In this way, three control models for SHMAX and two control models for AlexNet were trained that differed from the baseline model only in parameter λ for LAYER3, LAYER4, and LAYER5 in SHMAX and for LAYER2 and LAYER4 in AlexNet. We found that, in any layer, the PMI increased as the sparseness level increased (Figures 6B,C).

As mentioned before, the response sparseness in the baseline AlexNet is attributed to the rectified linear activation function. One would expect that changing the activation function to the sigmoid function would lead to less sparse activity in the model. A control model was constructed with this setting. With the aid of batch normalization (Ioffe and Szegedy, 2015), it was trained successfully on the ImageNet dataset (section Materials and Methods). With similar layer grouping, it was found that the sigmoid function led to much lower sparseness and PMI in the model compared with the rectified linear function (Figure S2). This result again supports the strong correlation between the sparseness level and PMI.

The above results did not imply that all units with higher lifetime sparseness tended to prefer CM images; in fact, many units with higher lifetime sparseness tended to prefer SM images. This result was valid not only across layers but also within the same layer. A scatter plot of the lifetime sparseness and the modulation index of the units in each layer of each model exhibited a “tornado” pattern: the units with lower sparseness were distributed within a narrower band in the modulation index axis centered at about zero, whereas the units with higher sparseness were distributed within a wider band in the modulation index axis (Figure 6D). Importantly, this pattern was not symmetric around zero but skewed to the positive side.

**Figure 7:**
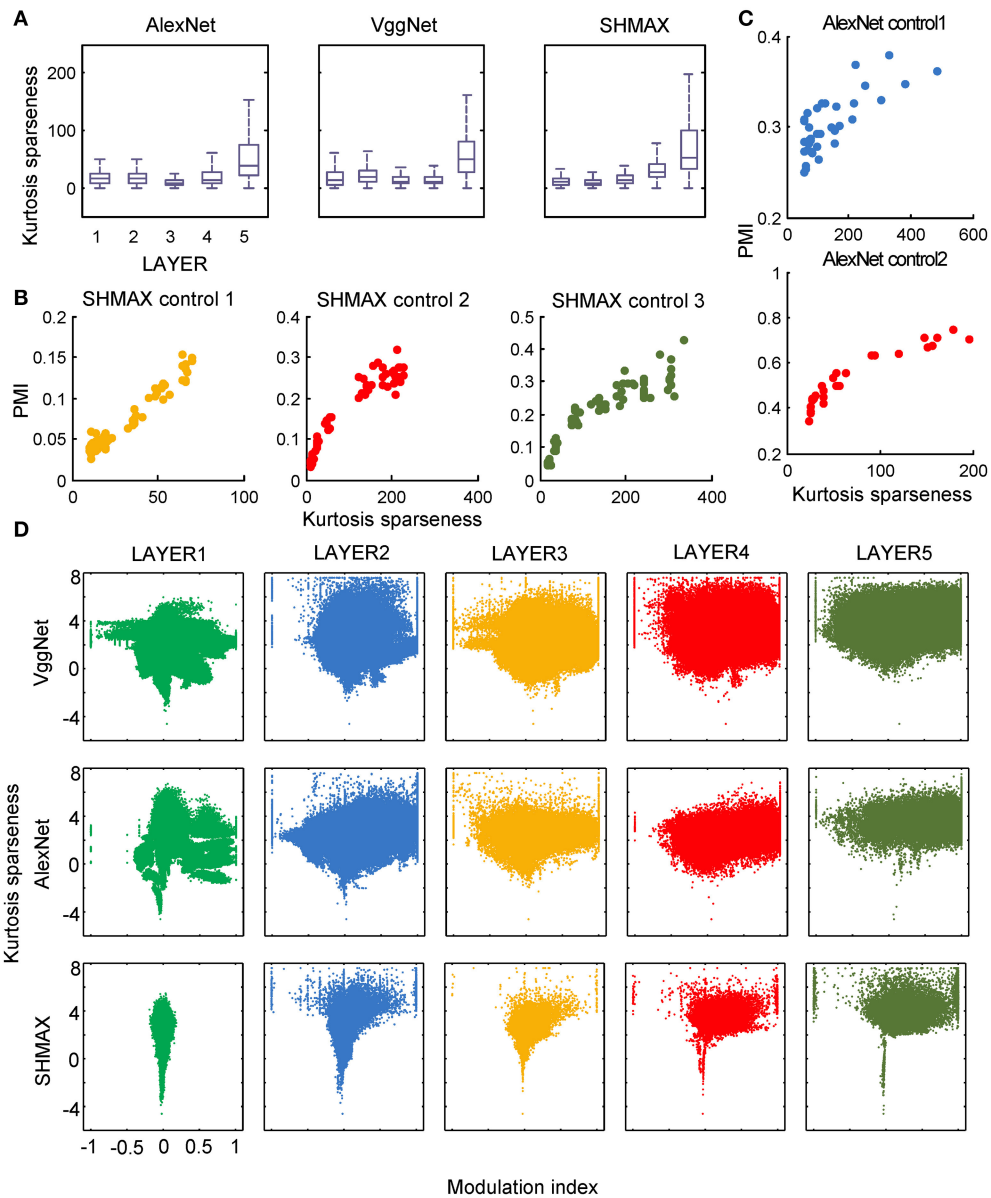
Relationship between the modulation and the response sparseness of units in the models measured by kurtosis. These data differ from Figure 6 only in the sparseness measure. In the y-axis of **(D)**, log (kurtosis) is used, and the minimum value of kurtosis is set to 0.01.

**Figure 8:**
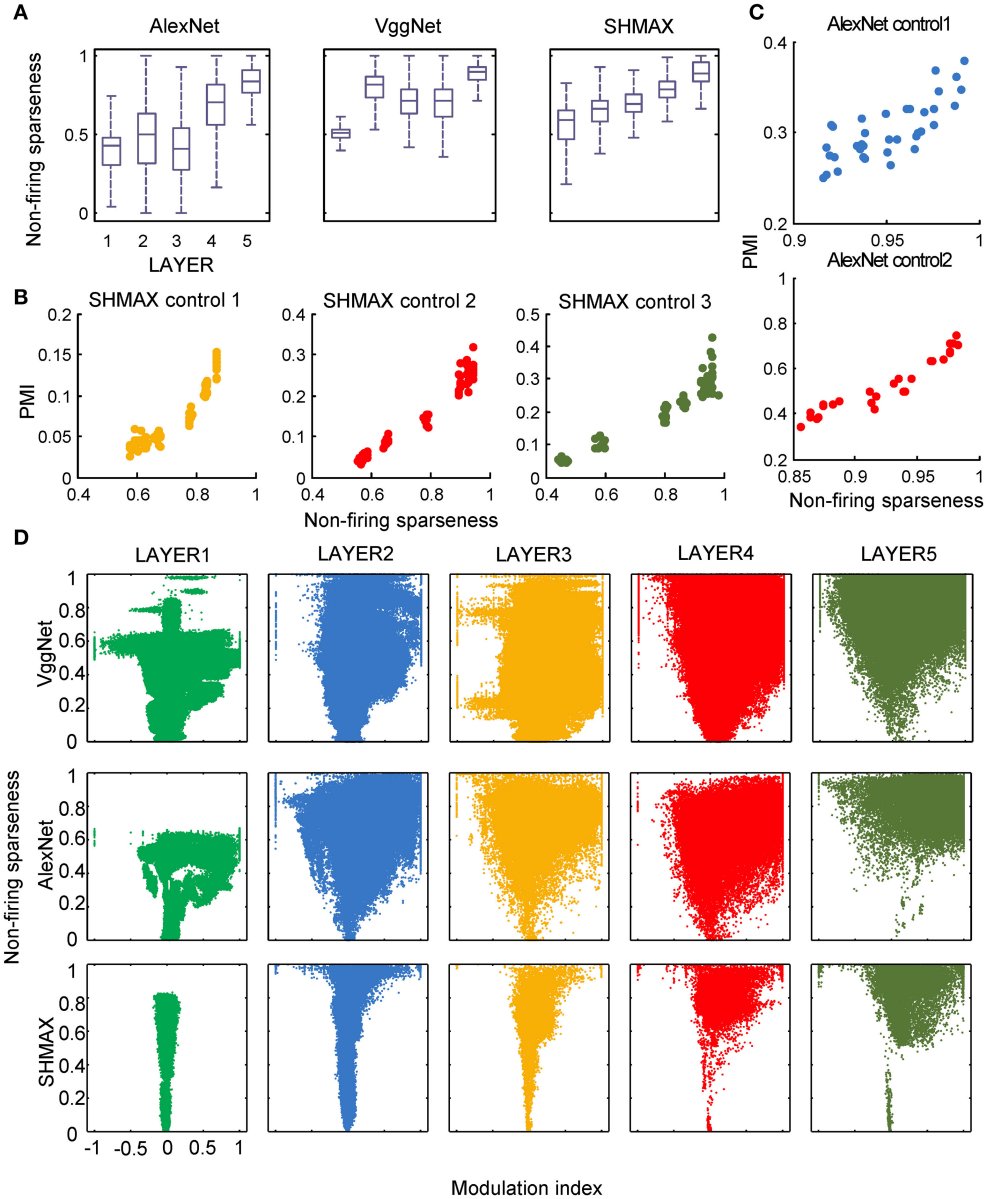
Relationship between the modulation and the response sparseness of units in the models measured by non-firing rate. These data differ from Figure 6 only in the sparseness measure.

**Figure 9:**
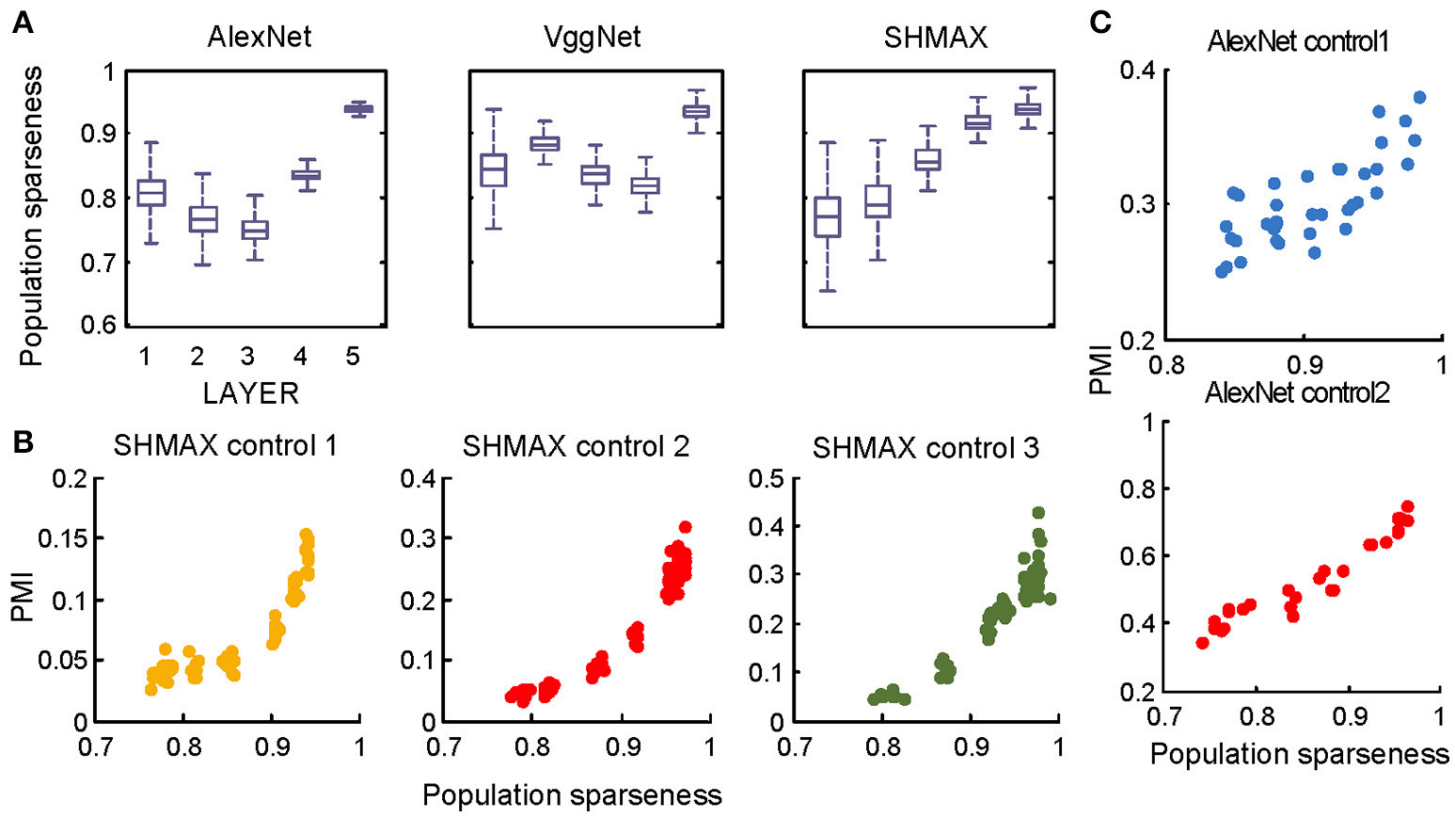
Relationship between the modulation and the response sparseness of units in the models measured by population sparseness. These data differ from Figures 6A–C only in the sparseness measure. Unlike in Figure 6, there is no panel **(D)** here because population sparseness is not defined for single units.

In the study of neuroscience, the term “sparseness” has several definitions, and the definitions may not correlate with one another (Willmore and Tolhurst, 2001; Willmore et al., 2011). Some definitions are for a single neuron responding to many stimuli (such as the lifetime sparseness definition used above), and others are for a population of neurons responding to a single stimulus. However, using different definitions of sparseness, including kurtosis, non-firing sparseness, and population sparseness (section Materials and Methods), we obtained qualitatively similar results to those observed using lifetime sparseness (Figures 7–9).

### Different receptive field sizes between layers do not explain the modulation difference

Neurons in higher areas of the visual cortex have larger receptive fields (RFs) on average, and it is possible that V2 neurons prefer CM images simply because their RFs contain more naturalistic structures than those of V1 neurons. However, this possibility was previously ruled out by showing no evidence for a correlation between RF size and modulation (Freeman et al., 2013). Because the effect of increasing RF size along the ascending hierarchy was also present in the computational models owing to the interleaving pooling layers, it is unknown if it was this factor that induced higher modulation in higher layers.

**Figure 10:**
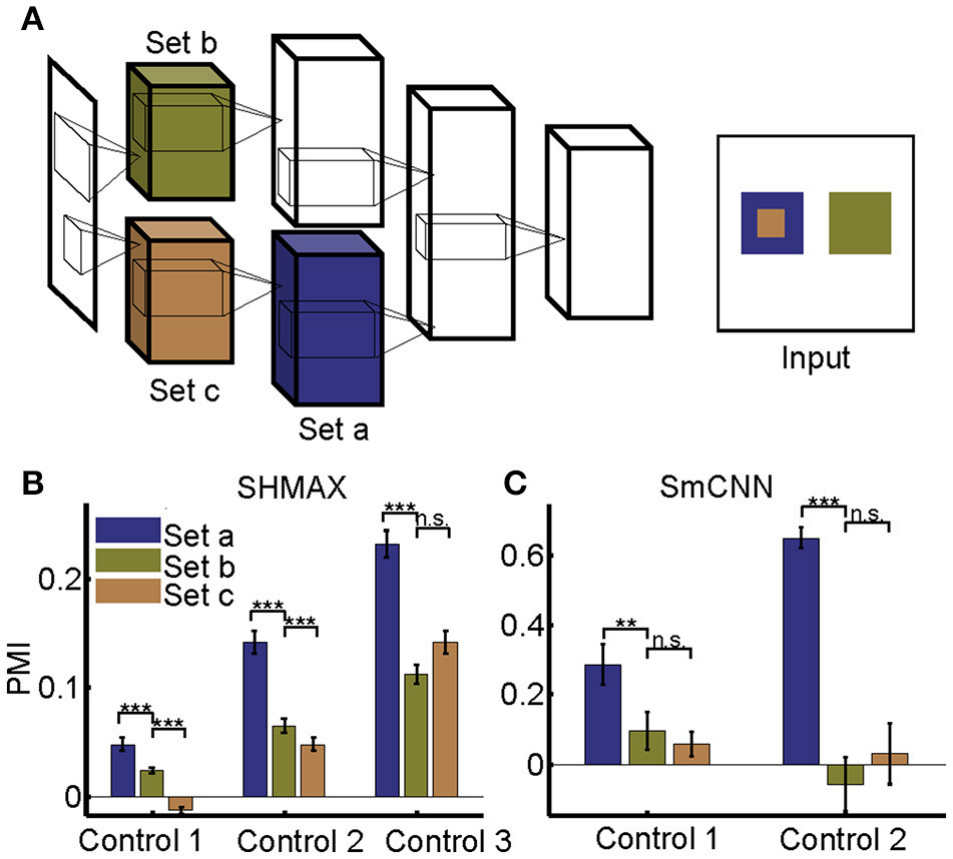
Effect of different RF sizes on modulation. **(A)** Illustration of the control models obtained by separating the feature maps of two consecutive layers of a baseline model into two sets (e.g., the lower layer is split into set *b* and set *c*) such that the RF size of units in set *a* is equal to that of units in set *b*, but larger than that of units in set *c*. The RF sizes are illustrated in the right panel. **(B,C)** PMIs of different sets of units (drawn in the same colors as in **(A)** in control models based on SHMAX and SmCNN. For SHMAX, controls 1–3 correspond to models with modifications in LAYER2 and LAYER3, LAYER3 and LAYER4, and LAYER4 and LAYER5, respectively. For SmCNN, controls 1 and 2 correspond to models with modifications in LAYER1 and LAYER2, and LAYER3 and LAYER4, respectively. The error bars indicate standard deviation over 10 models trained in the same way but from different initial values of parameters. Statistical analysis between set *a* and set *b* is one-tailed paired *t*-tests, and between set *b* and set *c* is two-tailed paired *t*-tests. ^**^P < 0.01; ^***^P < 0.001; n.s., not significant.

Different from their biological counterpart, these computational models have the same size RFs in the same layers, making it impossible to analyze the effect of RF size in the same way as it was analyzed in monkeys (Freeman et al., 2013). Our solution was to first construct a two-path deep learning model, such that the RF size in a given layer of one path was equal to the RF size in a different layer of the other path, and then to compare the modulation of the two layers (section Materials and Methods; Figure 10A).

We first tailored SHMAX in this manner. Because the PMI increased from LAYER2 to LAYER5 in the baseline model (Figure 2), we constructed three control models by manipulating the sizes of RFs for the units in neighboring layers, namely, LAYER2 and LAYER3, LAYER3 and LAYER4, and LAYER4 and LAYER5, respectively, in the three models and trained them with settings similar to those for the baseline model. The results of the first and second control models indicated that, within the same layer, units with larger RF sizes tended to have a larger modulation index (one-tailed paired *t*-test, *P* < 8.2 × 10^−6^). However, this result was not observed in the third control model. By contrast, in all control models, the PMI in the higher layer was much larger than that in the lower layer (one-tailed paired *t*-test, *P* < 5.4 × 10^−7^), despite the units in the two layers having the same size RFs (Figure 10B).

Tailoring AlexNet and VggNet for this purpose was difficult because they were big and hard to train. We therefore designed a small CNN with four big layers, LAYER1 to LAYER4 (section Materials and Methods), termed SmCNN, as the baseline model. After training on a quarter of the ImageNet Large Scale Visual Recognition Challenge 2012 (ILSVRC2012) dataset (Russakovsky et al., 2015), ~3.0 × 10^5^ images, the network exhibited increasing PMIs from the lower to higher layers, except between LAYER2 and LAYER3 (Figure 5). Therefore, we examined the influence of RF size by manipulating only LAYER1 and LAYER2 (control model 1) and LAYER3 and LAYER4 (control model 2). After training, we found that a larger RF size did not lead to a larger PMI for the computational units (Figure 10C). Instead, units in the higher layers had a larger modulation than those in the lower layers, although their RF sizes were the same.

Taken together, these results indicate that RF size differences cannot explain unit preference differences for naturalistic textures in different layers.

### Similarities between training images and CM images do not explain the difference in modulation

**Figure 11:**
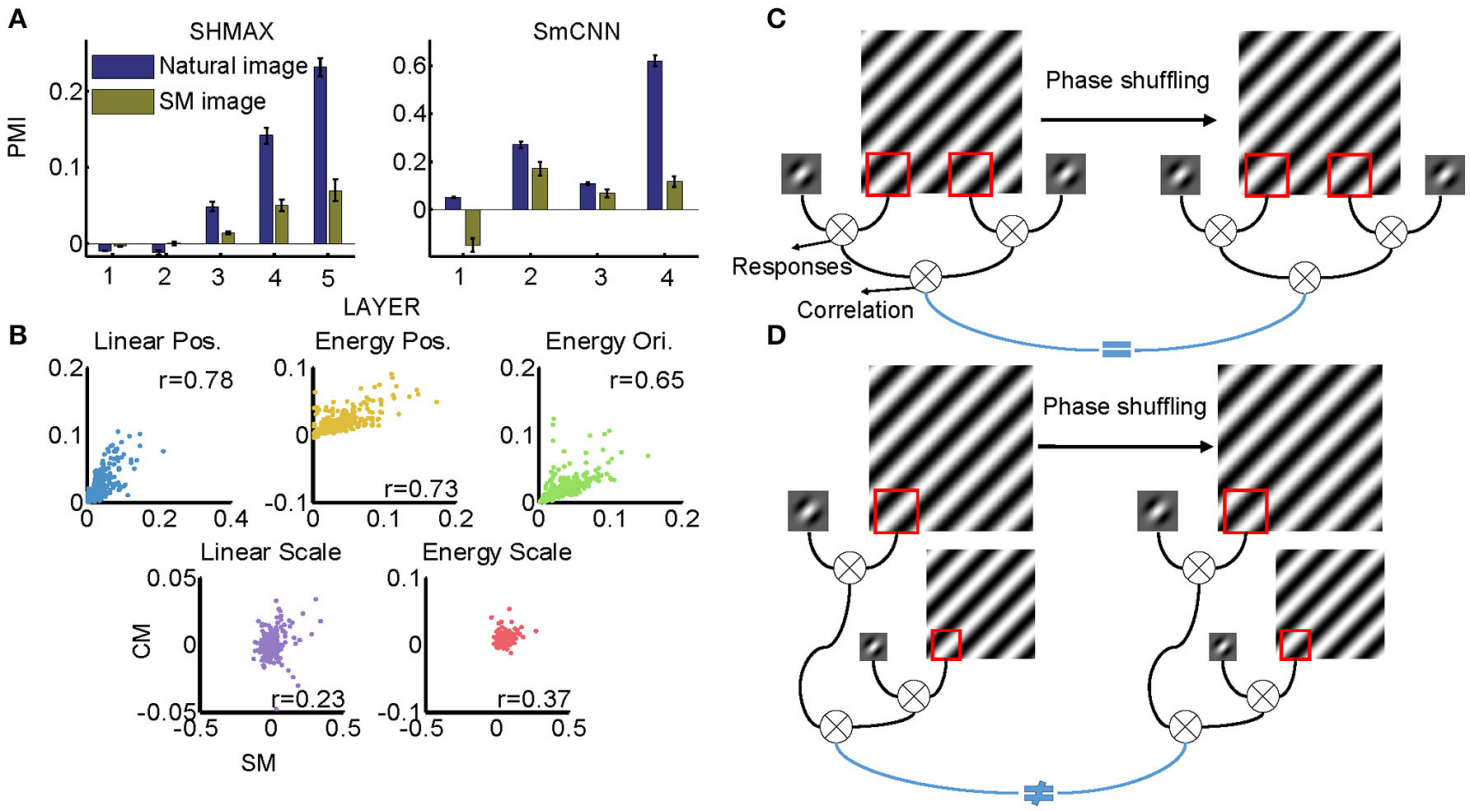
Effect of training images on modulation. **(A)** Comparison of PMIs in different layers of SHMAX and SmCNN trained on natural and SM images. The error bars indicate standard deviation over 10 trials with different initial parameters. **(B)** The correlations of different groups of statistics in CM and SM images. Each dot corresponds to a pair of images. **(C)** The cartoon illustrates why the position-related statistics in the CM and SM images were highly correlated. A 2D Fourier component of the original image is shown on the left, and, after phase shuffling, it becomes a component of the SM image, which is shown on the right. The Gabor filter represents a linear V1-like filter that was applied to the images to calculate responses. Correlations of these responses across locations on CM and SM images were highly correlated because they were insensitive to phase shuffling. **(D)** The cartoon illustrates why scale-related statistics in CM and SM images were less correlated. In calculating scale-related statistics, different Fourier components with different scales were used, with phases randomized independently, which led to less correlation of these statistics between CM and SM images.

All models were trained on natural images, leaving open the possibility that their higher layer units preferred CM images to SM images because the CM images looked more similar than the SM images did to the natural images. Thus, we next investigated whether the preference emerged when the models were trained on SM images. Because there were only 1,000 SM images, to avoid overfitting, we tested two small models, SHMAX and SmCNN. After training on these SM images, the higher layer units in both models exhibited a preference for CM images (Figure 11A), although the PMIs were smaller than those in the corresponding layers trained on natural images.

These results indicated that SM images contained certain higher-order statistics because otherwise the models could not have developed a preference in higher layers for CM images, which inherit many forms of higher-order statistics from the natural images. To investigate which groups of higher-order statistics were preserved in SM images, we projected correlations across position, scale, and orientation of linear filter responses or energies calculated on SM images and CM images to the corresponding principal components. We then visualized each group of statistics in pairs, with the SM image and CM image as two-dimensional points (Figure 11B). We found that the correlations of both linear filter responses and energy filter responses across different positions in the SM images were highly correlated with those in the CM images (Figure 11B, *r* = 0.7767 and 0.7297, respectively), indicating the presence of a certain amount of these statistics in SM images. This result was mainly because the correlation between responses of a filter at two fixed positions in the image plane was invariant to phase shuffling, which was used to generate SM images (Figure 11C).

However, not all types of statistics were preserved in SM images, for example, the correlations of both linear filter responses and energy filter responses across different scales, as these statistics in SM images and CM images showed low correlation (Figure 11B, 0.2308 and *r* = 0.3712, respectively). The reason for this can be explained as follows. In calculating this type of statistic, two filters (linear or energy) were separately applied to an image of two different scales, which were formed using different sets of Fourier components (Figure 11D). Consequently, the correlation of the two filters was sensitive to phase shuffling, a random operation for all Fourier components.

## Discussion

Recent studies show that, along the ventral visual pathway, higher areas, including areas V2 and V4, play more important roles than V1 for the perception of natural texture images (Freeman et al., 2013; Okazawa et al., 2015), but the mechanism underpinning this functional signature of the higher areas is unclear. In the present study, we first found this signature in higher layers of deep learning models and then revealed a strong correlation of this signature with response sparseness of the model neurons. Our findings suggest an important role for the sparse firing of neurons underlying the emergence of this signature in higher areas of the visual cortex.

Different forms of sparse neural firing have been experimentally observed in many areas of sensory cortices (Vinje and Gallant, 2000; Hromadka et al., 2008; Carlson et al., 2011; Willmore et al., 2011). From a metabolic perspective, sparse firing is energy efficient for neural encoding, as neurons do not respond vigorously to stimuli. From a computational perspective, this would reduce redundancy in the input such that a succinct neural code could be obtained (Barlow, 1989; Olshausen and Field, 1997). A number of studies support this function of sparse firing by showing that the outputs of computational models equipped with this characteristic match physiological results in visual cortex areas V1 (Olshausen and Field, 1996, 1997; Bell and Sejnowski, 1997) and V2 (Hosoya and Hyvarinen, 2015), but detailed comparative studies linking this function to physiological results in even higher areas are scarce. A computational model was previously proposed to fit object boundaries using a set of parametric curves that represent the RFs of V4 neurons (Carlson et al., 2011). The results of that study suggested that sparse firing underpinned the acute curvature preference of V4 neural responses. However, this single layer model is specific to V4 because it is built on the curvature representation of the V4 neurons. By contrast, our use of deep learning models enabled the simulation of all ventral pathway levels, and our results indicated a function of sparse firing in all higher layers.

Nevertheless, the following observations indicated that sparse firing was not the only factor contributing to this signature. First, models with similar response sparseness but random weights failed to exhibit this signature in higher layers (Figure 5). Although learning must have played an important role, the necessary conditions for successful learning remain unknown because both supervised and unsupervised learning led to the signature in our experiments. Second, the preference for naturalistic texture images in the bottom layers was significantly weaker than that in the higher layers (Figure 2), although bottom layers also exhibited response sparseness (Figures 6A, 7A, 8A, 9A). This observation highlights the importance of the hierarchical organization of the models.

**Figure 12:**
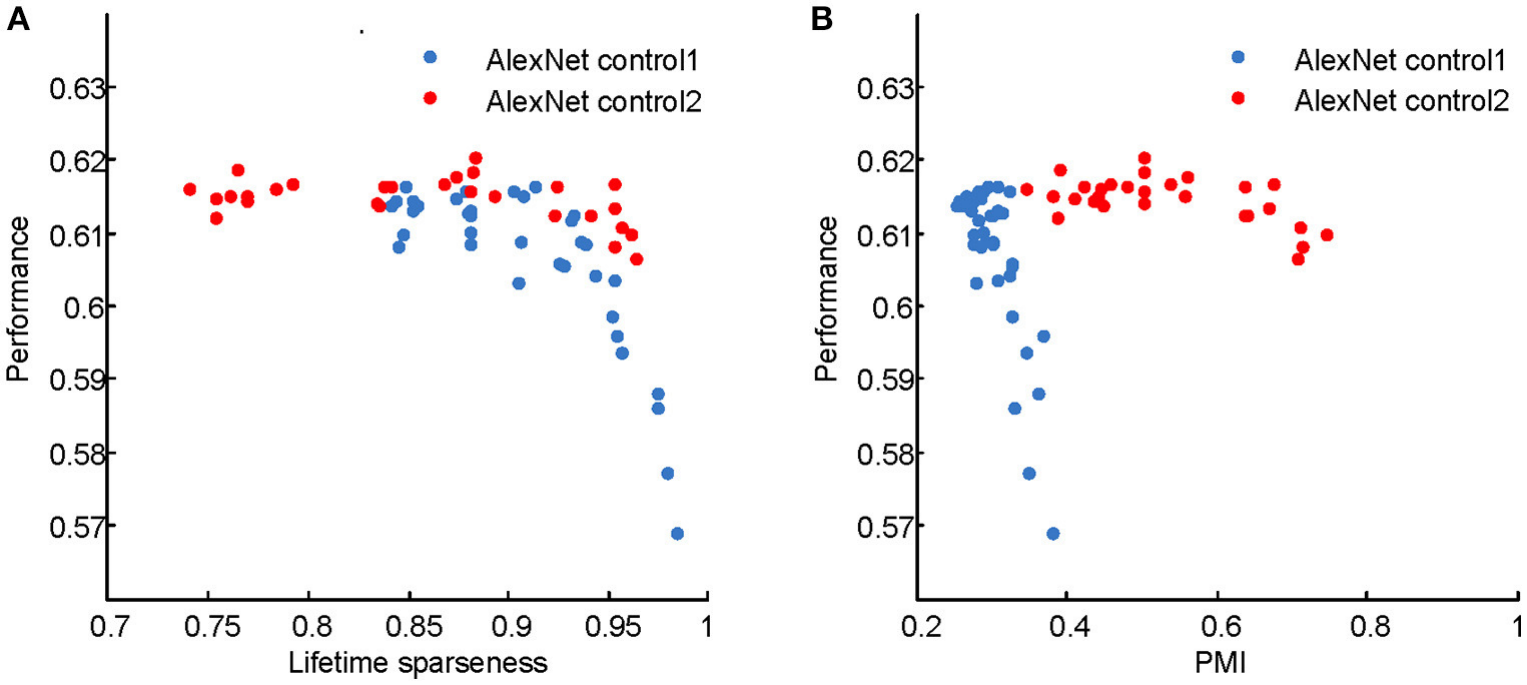
Classification accuracies of the AlexNet control models on the ImageNet test set. **(A)** Performance vs. PMI for AlexNet control 1 and AlexNet control 2. **(B)** Performance vs. lifetime sparseness for AlexNet control 1 and AlexNet control 2. For control model 1, with higher PMI, the performance decreases. For control model 2, within a wide range of PMI, the performances are similar. These results indicate that high PMI is not a sign of a better model in terms of classification performance.

The computational models used in this study are deep learning models, which originated in neuroscience but do not faithfully copy the structure of the brain. These models have recently gained success in various engineering applications, including image classification (Krizhevsky et al., 2012), speech recognition (Dahl et al., 2012), natural language processing (Sutskever et al., 2014), and game playing (Mnih et al., 2015; Silver et al., 2016). The neuroscience community has begun to investigate the link between deep learning models and the brain. Most of these studies aimed to reveal how well the models match the monkey's visual system by either fitting or comparing real neuronal responses in specific areas, such as V4 and the inferior temporal cortex, with the responses of the model neurons (Cadieu et al., 2007; Khaligh-Razavi and Kriegeskorte, 2014; Yamins et al., 2014), or comparing performances on certain tasks based on real and model neuronal responses (Cadieu et al., 2014). Different from those studies, we aimed to reveal the computational principles of the visual system based on deep learning models by manipulating their architecture, hyperparameters, and learning principles. According to Marr and Poggio's tri-level hypothesis (Marr and Poggio, 1977; Marr, 1983), it is possible that computational models share certain components with the brain at the computational theory and algorithmic levels, especially when the models robustly reproduce results measured in the brain, as in the present study. The common components for visual information processing suggested by the present study include hierarchical structure, response sparseness, and certain types of learning (Marblestone et al., 2016). Different types of learning correspond to optimizing different cost functions. It is hypothesized that the brain can optimize diverse cost functions (Marblestone et al., 2016). However, since both supervised and unsupervised learning led to qualitatively similar results in our experiments, we were unable to distinguish which cost function, prediction error or reconstruction error, plays a more important role in shaping the visual system during development. Recent studies emphasize the role of prediction error by fitting the activity of the deep learning model neurons to that of cortical neurons (Khaligh-Razavi and Kriegeskorte, 2014; Yamins et al., 2014; Yamins and DiCarlo, 2016). It is tempting to hypothesize that the functional signature found in higher visual areas is positively correlated with the classification performance of animals, but this was not validated in our deep learning models (Figure 12), although optimizing the latter led to the emergence of the former. These results indicate a complicated relationship between neural signature and behavioral performance.

The models generated some predictions testable in animals and humans. First, they predicted increasing modulation along the visual ventral pathway, although this trend was not perfect (Figure 2). Second, they predicted a “tornado” pattern for the distribution of neurons in any area along the ventral pathway in the modulation–sparseness plane (Figures 6D, 7D, 8D); that is, with higher response sparseness, neurons show greater preference for either CM images or SM images. Third, they predict a positive correlation between response sparseness and modulation of neurons in higher visual areas (Figures 6–9). Verification of this last prediction will require manipulating the activity level of neurons *in vivo*, which is technically difficult at present, but using certain types of microbial opsins in animals may be a solution (Atallah et al., 2012).

The limitation of the present study is obvious owing to the great difference between the computational models and the biological vision system. First, a real neuron has about 1,000 synapses but most model neurons in the convolutional layers (for CNN) or sparse coding layers (for SHMAX) have no more than 25 connections. Second, a large body of literature has reported anatomic difference in different visual cortical areas. For example, along the ventral pathway, starting from V1, neuron density decreases (Wilson and Wilkinson, 2015) while the number of dendritic spines of layer III pyramidal neurons increases (Elston and Rosa, 1998; Elston, 2002). However, the spatial arrangement and the shape of the neurons are not considered in these models. Third, both within areas and across areas recurrent synapses are abundant in the visual cortex (Dayan and Abbott, 2001; Gilbert and Li, 2013), but the models are purely feedforward architectures. It is unclear how these differences could influence the functional signature found in higher layers of the models. More biologically detailed models are entailed to answer this question. Nevertheless, devising such models is still a challenging problem in the deep learning community.

## Materials and methods

### Stimuli synthesis

The stimuli were generated using the same method described in two previous studies (Portilla and Simoncelli, 2000; Freeman et al., 2013). For each natural texture image, two images were synthesized, and these were called the SM and CM images. The SM image was synthesized by first computing the Fourier transform of the original image, then randomizing the phases of the Fourier components, and finally computing the inverse Fourier transform. This procedure is thought to preserve the spectral properties of the original image, such as the spatial-frequency content, and destroy higher-order statistics, such as the correlations between linear filter responses in different scales of the original image (Freeman et al., 2013), although our analysis suggested that a certain amount of the higher-order statistics were still preserved (Figure 11). The CM image was synthesized from Gaussian noise using an iterative procedure “to match the spatially averaged filter responses, the correlations between filter responses, and the mean, variance, skewness, and kurtosis of the pixel luminance distribution (‘marginal statistics’)” (Freeman et al., 2013) of the original image.

The original texture images were from a dataset (Lazebnik et al., 2005) consisting of 25 texture families, with 40 images per family. All images were resized from 640 to 480 pixels to 128 × 128 pixels to generate 1,000 SM images and 1,000 CM images of the same size, using companion codes of reference (Portilla and Simoncelli, 2000) with default settings. They were subtracted by their mean and resized to 224 × 224 pixels before being sent to the deep learning models, as the models were trained with images of this size.

### Computational models

Four deep learning models were used in the experiments. AlexNet (Krizhevsky et al., 2012) is a CNN, which has five convolutional layers (the number of filters for the five layers is 96, 256, 384, 384, and 256, respectively), interleaved with max pooling layers and local response normalization (LRN) layers. Each layer consists of a set of feature maps. A feature map of a convolutional layer is an ensemble of the responses of a filter on the output of the preceding layer. A max pooling layer or LRN layer has the same number of feature maps as its preceding layer. These layers were grouped into five big layers in the bottom-up direction (Figure 1B), named LAYER1 to LAYER5, each starting with a convolutional layer and ending with the preceding layer of the next convolutional layer. Therefore, the number of feature maps in each big layer was a multiple of the number of filters in the corresponding convolutional layer. VggNet (Simonyan and Zisserman, 2015) is a deeper CNN having 19 convolutional layers separated by four max pooling layers into five groups. The five groups, separated by four max pooling layers, each consisting of two to four consecutive convolutional layers, were named LAYER1 to LAYER5. The numbers of filters in the five layers were 64 × 2, 128 × 2, 256 × 4, 512 × 4, *and* 512 × 4, respectively, where the first number is the number of filters in a convolutional layer and the second number is the number of convolutional layers in the corresponding big layer. Both AlexNet and VggNet have some fully connected layers and an output layer; however, these layers were not investigated in this study because their structures differ significantly from that of the convolutional layers, pooling layers, and LRN layers. For fast training in an experiment (Figure 10), a small CNN, termed SmCNN, was designed. It was obtained by deleting LAYER5 in AlexNet and decreasing the number of filters in the lower layers and the number of units in the fully connected layers. The numbers of filters in LAYER1 to LAYER4 were 64, 192, 160, and 128, respectively. The numbers of hidden units in fully connected layers were all 2048. The activation function in AlexNet and VggNet is the rectified linear function *f*(*x*) = *max*(*x*, 0). SHMAX (Hu et al., 2014) is a deep learning model consisting of alternating sparse coding layers and max pooling layers. A SHMAX with a similar architecture to the first five big layers of AlexNet was designed by deleting the LRN layers and substituting the convolutional layers with sparse coding layers. The pooling layers were the same as those in AlexNet, including pooling sizes and strides.

AlexNet and VggNet were trained on 1.2 million images in 1,000 classes from the ILSVRC2012 dataset (Russakovsky et al., 2015). The models were directly tested using the pre-trained weights downloaded from the website of MatConvNet (Vedaldi and Lenc, 2015). SmCNN was trained on one-fourth of the dataset using Cuda-convnet2 (Krizhevsky et al., 2012). The performance of the model for classification was satisfactory (top-1 error rate 59.128% and top-5 error rate 34.156% on ~5.0 × 10^4^ test images).

SHMAX was trained by layer-wise sparse coding with the constraint that unit responses were non-negative.

(1) $$\begin{array}{l} \text{ minimize }\sum_{j=1}^{K}\left({\left|\left|x_{j}-As_{j}\right|\right|}_{2}^{2}+\lambda {\left|\left|s_{j}\right|\right|}_{1}\right)\\ \text{subject to }{\left|\left|a_{i}\right|\right|}_{2}^{2}\leq 1,\;s_{ij}\geq 0,\;\forall i=1,\ldots ,M;j=1,\ldots ,K, \end{array}$$

where **x**_*j*_ is an input (image patch for the first convolutional layer or feature patch for other convolutional layers), each column of **A**, denoted by **a**_*i*_, is a basis, and **s**_*j*_ is the coefficient vector, which can be regarded as responses of *M* units to the input **x**_*j*_. The parameter λ in the objective function controls the balance of the reconstruction error (the first term) and the level of population sparseness (the second term). Unless otherwise stated, parameter λ was set to 0.15 for LAYER1 and LAYER2, and to 0.1 for LAYER3 to LAYER5. Training this model with a large dataset was technically difficult because it demanded a huge memory. Therefore, 1.0 × 10^4^ images were randomly chosen from the ILSVRC2012 dataset as training images. To learn the bases for the current layer, for every training image, 200 patches of the same size were randomly selected from this layer.

### Calculating the modulation index

For each layer, every element in every feature map was treated as a model “neuron,” or unit, in that layer. For example, in the first convolution layer of AlexNet, there were 55 × 55 × 96 = 290400 units, where the first two numbers corresponded to the dimensions of the feature map and the third number corresponded to the number of filters.

In CNN, the response of a unit was the value after the linear rectifier activation function, which was always non-negative. In SHMAX, the unit response was calculated according to Equation (1) based on learned bases **A**, which was also non-negative. The modulation index of a unit was defined as the difference in its responses to the CM and SM image pair generated from the same natural image divided by their sum, then averaged over all CM–SM pairs. If the unit did not respond to either the CM image or the SM image in a CM–SM pair, then this pair was excluded in calculating the modulation index for the unit. The PMI was defined as the mean modulation index of a set of units, for example, all units in a layer of a model, with respect to a set of images. If a unit did not respond to any image in the dataset, it was excluded in calculating the PMI. Unless otherwise indicated, PMI was calculated over all CM–SM pairs across all texture families.

### RD between two sequences of orders

Let *X* and *Y* denote two sequences of orders (two permutations of 1–*n*). For every number *x*_*i*_ in *X*, denote its index in *Y* by *f*_*Y*_(*x*_*i*_). RD between *X* and *Y* is defined as

(2) $$D_{n}\left(X,Y\right)=\sum_{i=1}^{n}\left|\log\left(\frac{i}{f_{Y}\left(x_{i}\right)}\right)\right|.$$

It can be proved that RD is a valid distance. To show this, the distance defined in Equation (2) must satisfy the following conditions:

1. *D*_*n*_ (*X, Y*) ≥ 0 (non-negativity)
2. *D*_*n*_ (*X, Y*) = 0 ⇔ *X* = *Y* (identity of indiscernibles)
3. *D*_*n*_ (*X, Y*) = *D*_*n*_ (*Y, X*) (symmetry)
4. *D*_*n*_ (*X, Z*) ≤ *D*_*n*_ (*X, Y*) + *D*_*n*_ (*Y, Z*) (triangle inequality),

where *X, Y* are two arbitrary permutation sequences of 1–*n*.

It is obvious that the first condition holds. The second condition is proved as follows.

- If *D*_*n*_ (*X, Y*) = 0, then for all *i* ∈ {1, 2, …, *n*}, $\log\left(\frac{i}{f_{Y}\left(x_{i}\right)}\right)=0$ and $\frac{i}{f_{Y}\left(x_{i}\right)}=1$, which indicates *x*_*i*_ = *y*_*i*_ according to the definition of *f*_*Y*_ (*x*_*i*_). In other words, *X* = *Y*.
- If *X* = *Y*, obviously *D*_*n*_ (*X, Y*) = 0.

The third condition holds because of the following:

$$\begin{array}{l} D_{n}\left(X,Y\right)=\sum_{i=1}^{n}\left|\log\left(\frac{i}{f_{Y}\left(x_{i}\right)}\right)\right|=\sum_{i=1}^{n}\left|\log\left(\frac{f_{Y}\left(x_{i}\right)}{i}\right)\right|\\ \;=D_{n}\left(Y,X\right). \end{array}$$

In the above reasoning, we used the fact that {*f*_*Y*_(*x*_1_), *f*_*Y*_(*x*_2_), …, *f*_*Y*_(*x*_*n*_)} is also a permutation sequence of 1 – *n*. The fourth condition is proved as follows.

$$\begin{array}{l} D_{n}\left(X,Y\right)+D_{n}\left(Y,Z\right)=\sum_{i=1}^{n}\left|\log\left(\frac{i}{f_{Y}\left(x_{i}\right)}\right)\right|+\sum_{i=1}^{n}\left|\log\left(\frac{i}{f_{Z}\left(y_{i}\right)}\right)\right|\\ \;=\sum_{i=1}^{n}(\left|\log\left(\frac{i}{f_{Y}\left(x_{i}\right)}\right)\right|+\left|\log\left(\frac{f_{Y}\left(x_{i}\right)}{f_{Z}\left(y_{f_{Y}\left(x_{i}\right)}\right)}\right)\right|)\\ \;\geq \sum_{i=1}^{n}\left|\log\left(\frac{i}{f_{Y}\left(x_{i}\right)}\right)+\log\left(\frac{f_{Y}\left(x_{i}\right)}{f_{Z}\left(y_{f_{Y}\left(x_{i}\right)}\right)}\right)\right|\\ \;=\sum_{i=1}^{n}\left|\log\left(\frac{i}{f_{Y}\left(x_{i}\right)}\cdot \frac{f_{Y}\left(x_{i}\right)}{f_{Z}\left(y_{f_{Y}\left(x_{i}\right)}\right)}\right)\right|\\ \;=\sum_{i=1}^{n}\left|\log\left(\frac{i}{f_{Z}\left(x_{i}\right)}\right)\right|=D_{n}\left(X,Z\right). \end{array}$$

In the above reasoning, we used the facts: *y*_*f*_*Y*_(*x*_*i*_)_ = *x*_*i*_, {*f*_*Y*_(*x*_1_), *f*_*Y*_(*x*_2_), …, *f*_*Y*_(*x*_*n*_)} is a permutation sequence of 1 – *n*, and |*x*| + |*y*| ≥ |*x* + *y*|. Therefore, RD is a valid distance (or metric). The smaller the RD between two sequences, the more consistent the sequences are.

The permutation test can be used to determine whether two sequences are consistent. First, a large number of random permutations of 1–*n* are generated. The RD values between them are then calculated. These distances constitute a distribution of the null hypothesis that two sequences are inconsistent. The percent of distances in the distribution smaller than the distance between the two tested sequences is the *P*-value of the test.

### Fitting the modulations of top layer units using image statistics

The aim here was to predict the PMIs in LAYER5 of the models to a pair of CM–SM images based on the statistics of the corresponding natural image used to generate the CM image (Figure 4). The statistics of each image consisted of 1,104 parameters, which were grouped as follows (Freeman et al., 2013; Okazawa et al., 2015): (1) marginal statistics (including skewness and kurtosis); (2) spectral statistics (average energy in sub-bands); (3) correlations of linear filter responses at neighboring locations; (4) correlations of linear filter responses at neighboring scales; and (5) correlations of energy filter responses at neighboring orientations, (6) neighboring locations, and (7) neighboring scales. Each parameter was transformed by taking its signed square root followed by z-score normalization such that its mean was zero and its standard deviation was one (Freeman et al., 2013). The number of parameters was too large for predicting a set of unit PMIs with respect to a pair of CM–SM images, as there were only 1,000 image pairs. Principal component analysis (PCA) was then performed on different groups of parameters separately, and the first several components were selected to cover more than 90% of the variance, usually 4–12 components. Finally, 74 parameters were obtained that made linear fitting feasible (Figure 4A).

To compute the contributions of different groups of parameters to the PMI, a procedure known as averaging-over-orderings was followed (Gromping, 2007). The contribution of a particular group of parameters was measured by the difference in *R*^2^ of the linear fitting between a model with this group of parameters and a model without it. Since the difference depended on the order in which this group of parameters was added, differences for all possible orders of additions were computed and the results were averaged to obtain the final contribution. The averaged difference was divided by the *R*^2^ of the full model to obtain the percentage contribution of this group of parameters.

### Calculation of response sparseness

Four types of unit response sparseness were calculated based on the responses of the units to 2000 images randomly selected from the ILSVRC2012 dataset (Russakovsky et al., 2015). The definition of the lifetime sparseness of a unit was as follows (Willmore et al., 2011):

(3) $$S=1-\frac{{\left(E[r]\right)}^{2}}{E[r^{2}]},$$

where the expectation was taken across all test images and *r* denotes the response of the unit. The non-firing sparseness of a unit was simply the frequency with which that unit did not respond. The lifetime kurtosis of a unit was the fourth standardized moment across its response to all natural images (Vinje and Gallant, 2000). Unlike the aforementioned three types of sparseness, which were defined for single units, population sparseness was defined for a population of units, usually all units in one layer of a deep learning model. For each input image, it was calculated according to Equation (3), but the expectation was taken across all units (Willmore and Tolhurst, 2001).

### Changing sparseness of SHMAX

For each of the three control experiments (Figures 6B, 7B, 8B, 9B), we only changed the sparseness of a particular layer, that is, LAYER3, LAYER4, or LAYER5. This was achieved by setting different λ values in equation (1) for sparse coding in the present big layer (λ was fixed at default values in preceding layers). In the experiments, 0.01, 0.02, 0.05, 0.1, 0.25, 0.35, and 0.45 were used for λ. For every setting, 10 models were trained starting from different initial values.

### Changing sparseness of alexnet

To control the population response sparseness of units in the *j*-th layer of AlexNet, a regularization term was added to the original loss function *L*_*orig*_

(4) $$\text{minimize }L_{orig}+\lambda {\left|\left|r_{j}\right|\right|}_{1},$$

where **r**_*j*_ denotes the responses of units in the *j*-th convolution layer after the linear rectifier activation function, and λ is a balancing parameter. In two control experiments (Figures 6C, 7C, 8C, 9C), the sparseness of LAYER 2 and then LAYER 4 was changed. For LAYER 2, λ varied among {0.01, 0.02, 0.05, 0.1, 0.2, 0.5, 1, 2, 10} × 10^−10^; and, for LAYER 4, it varied among {0.01, 0.03, 0.1, 0.3, 1, 3, 10} × 10^−10^. For every setting, five models were trained starting from different initial values. The classification performances of these sparseness-controlled models on the ImageNet dataset are presented in Figure 12.

The sparse activity in the baseline AlexNet is mainly introduced by the rectified linear activation function. A control model was constructed by replacing this function in AlexNet with the sigmoid activation function $f\left(x\right)=\frac{1}{1+exp\left(-x\right)}$, which does not introduce as much sparse activity as the rectified linear function. But training such a model on the ImageNet dataset is difficult due to the notorious gradient vanishing effect (Hochreiter, 1991). This difficulty is alleviated by adding a batch normalization layer (Ioffe and Szegedy, 2015) after each convolution layer. Five models were trained starting from different initializations and each of them achieved roughly 51% of top-1 error rate.

### Changing RF size

To investigate the effect of RF size on a baseline (single-chain) model, the feature maps of two consecutive layers were separated into two sets (e.g., the lower layer was split into set *b* and set *c*) and two parallel paths in these layers were constructed (Figure 10A). Different kernel sizes were used in these sets such that the RF size of units in set *a* was equal to that of units in set *b*, but larger than that of units in set *c*, as illustrated in Figure 10A (right). Paddings were used to ensure that the two sets of feature maps in the second stage of the parallel paths were of the same size, which was necessary for constructing subsequent layers. This approach was applied to three pairs of layers in SHMAX, namely, LAYER2 and LAYER3, LAYER3 and LAYER4, and LAYER4 and LAYER5, and two pairs of layers in SmCNN, i.e., LAYER1 and LAYER2, and LAYER3 and LAYER4. Other settings and the training schemes remained the same as those for the baseline models.

### Statistical testing

Except where noted, all statistical tests for the differences of modulation in two conditions were one-tailed unpaired *t*-tests. Because each layer of the models had a large number of units (usually hundreds of thousands), trivial differences between two layers would become significant using a standard *t*-test. To rectify this problem, a random sampling *t*-test (RST) approach was employed. For comparing the mean modulation indices of two groups of units (Figure 2, left) or the mean modulation index of one group with zero (Figure 2, right), 100 units from the groups were repeatedly sampled 500 times and standard *t*-tests were performed each time; then the *P*-values were averaged to obtain the final *P* value. This random sampling procedure simulated electrode recordings in the brain.

Analysis of the significance of the modulation for each unit (Figure 2, left, red curves) was computed using a randomization test (Freeman et al., 2013). The labels of all CM images and SM images were randomly shuffled, and the modulation index of each unit was computed. This procedure was repeated 1 × 10^4^ times. Then, the fraction of the resulting null distribution that was larger than the original modulation index for that unit was computed. If this fraction was smaller than 0.05, the unit showed a significant positive modulation index.

## Author contributions

CZ and XH designed the experiments. CZ and YW conducted the experiments. CZ, XH, and DY analyzed the data and wrote the paper. All authors read and approved the final manuscript.

### Conflict of interest statement

The authors declare that the research was conducted in the absence of any commercial or financial relationships that could be construed as a potential conflict of interest.
