# Supplementary material for: Deep Learning Predicts Correlation between a Functional Signature of Higher Visual Areas and Sparse Firing of Neurons
Source: Front Comput Neurosci. 2017 Oct 30;11:100. doi: 10.3389/fncom.2017.00100 (PMC5670118; doi:10.3389/fncom.2017.00100)
Supplement: Supplementary file 1 [file Presentation1.PDF]

Supplementary Figures

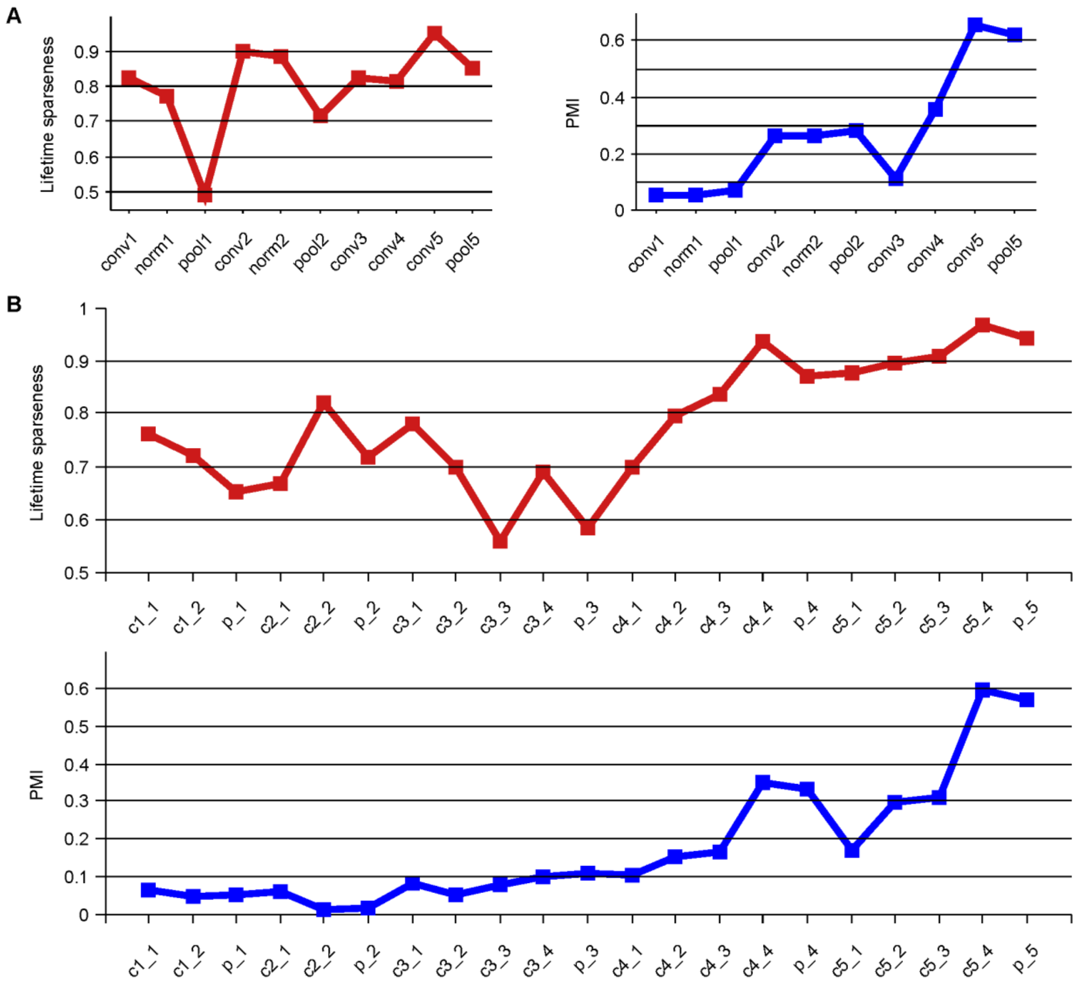

Figure S1 | The lifetime sparseness and PMI of AlexNet (A) and VggNet (B) in their original layers. In (**B**) "cN_M" denotes the M-th convolution layer in LAYER N and "p_N" denotes the pooling layer in LAYER N, which is also the N-th pooling layer in VggNet.

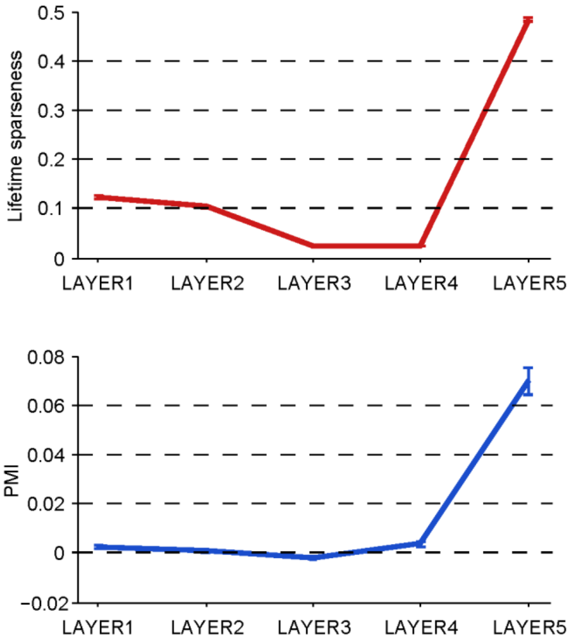

Figure S2 | The lifetime sparseness (blue) and PMI (red) of AlexNet using the sigmoid function as the activation function. The error bars indicate the standard deviation of 5 models with the same structure starting from different initial weights.
